# New phase therapeutic pursuits for targeted drug delivery in glioblastoma multiforme

**DOI:** 10.37349/etat.2022.00118

**Published:** 2022-12-30

**Authors:** Manisha Singh, Divya Jindal, Vinayak Agarwal, Deepanshi Pathak, Mansi Sharma, Pranav Pancham, Shalini Mani, Rachana

**Affiliations:** Department of Biotechnology, Jaypee Institute of Information Technology (JIIT), Noida 201301, India; Université Paris-Saclay, France

**Keywords:** P53 pathway, retinoblastoma pathway, receptor tyrosine kinase (RTK)/rat sarcoma virus (Ras)/phosphoinositide 3-kinase (PI3K) pathway, theranostic biomarkers, immune therapy, gene therapy

## Abstract

Glioblastoma multiforme (GBM) is known as the most aggressive and prevalent brain tumor with a high mortality rate. It is reported in people who are as young as 10 years old to as old as over 70 years old, exhibiting inter and intra tumor heterogeneity. There are several genomic and proteomic investigations that have been performed to find the unexplored potential targets of the drug against GBM. Therefore, certain effective targets have been taken to further validate the studies embarking on the robustness in the field of medicinal chemistry followed by testing in clinical trials. Also, The Cancer Genome Atlas (TCGA) project has identified certain overexpressed targets involved in the pathogenesis of GBM in three major pathways, i.e., tumor protein 53 (p53), retinoblastoma (RB), and receptor tyrosine kinase (RTK)/rat sarcoma virus (Ras)/phosphoinositide 3-kinase (PI3K) pathways. This review focuses on the compilation of recent developments in the fight against GBM thus, directing future research into the elucidation of pathogenesis and potential cure for GBM. Also, it highlights the potential biomarkers that have undergone extensive research and have promising prognostic and predictive values. Additionally, this manuscript analyses the advent of gene therapy and immunotherapy, unlocking the way to consider treatment approaches other than, or in addition to, conventional chemo-radiation therapies. This review study encompasses all the relevant research studies associated with the pathophysiology, occurrence, diagnostic tools, and therapeutic intervention for GBM. It highlights the evolution of various therapeutic perspectives against GBM from the most conventional form of radiotherapy to the recent advancement of gene/cell/immune therapy. Further, the review focuses on various targeted therapies for GBM including chemotherapy sensitization, radiotherapy, nanoparticles based, immunotherapy, cell therapy, and gene therapy which would offer a comprehensive account for exploring several facets related to GBM prognostics.

## Introduction

Over the past century, therapeutic approaches for cerebral malignant tumors have remained a critical challenge. Though after several refinements in various techniques that range from neurosurgical options to the breakthrough of effective chemotherapeutic agents, improvements in radiotherapy, biotechnological advancements for targeted delivery, etc., have led to an extended and improvised survival in patients suffering from many other types of brain tumors, except glioblastoma multiforme (GBM) [1]. It is still one of the most communal and malignant tumors in adults, covering almost 16% of primary brain and central nervous system (CNS) diseases. The average aged occurrence rate for GBM is 3.2 per 100,000 people in the population [2]. Even though GBMs happen solely in the brains, they can likewise show up in the cerebrum stem cells and spinal cord too. It’s been reported that around 61% of essential gliomas of the brain exist in the four major projections: front (25%), transient (20%), parietal (13%), and occipital (3%) [3]. However, initially, GBMs were believed to be obtained exclusively from glial cells, nonetheless, later many studies reported that these gliomas might emerge from various cell types comprising neural stem cell-like behaviour and properties.

Furthermore, GBMs are classified as primary, or *de novo* types, as they get developed without a known precursor, or secondary ones when a low-grade tumor develops into a GBM over time. Most of the GBMs are primary, and patients with primary GBMs are older and have a worse prognosis than those with secondary forms of GBMs. Earlier, GBMs were assumed to develop from glial cells, however, the research data in the last decade and a half reveals that they can come from a variety of cell types with neural stem cell-like features [4]. As we know that malignant cells are in various stages of differentiation, whether it is stem cells, neurons, or glial cells, they are marked by certain phenotypic distinctions largely. And these distinctions are further defined by molecular changes in the signaling pathways instead of differences in the origin of the cells [5]. GBMs are found to be more common in those over the age of 60 years and it has been observed that men have a little greater reported incidences than women, while Caucasians have a slightly higher probability than other ethnic groups [6].

Subsequently, with more research initiatives from the global scientific communities, until now over 600 specific genes have been sequenced from 200 human tumors in genome profiling and the Disease Genome Map Book Project. This project has confounded the hereditary profile of GBM and provided three important signaling pathways that are involved in GBM tumor protein 53 (p53), retinoblastoma (RB), and receptor tyrosine kinase (RTK)/rat sarcoma virus (Ras)/phosphoinositide 3-kinase (PI3K) pathways [7]. The majority of GBMs (primary and secondary) had mutations in these pathways, allowing tumor cells to evade cell-cycle checkpoints, senescence, and apoptosis pathways, resulting in hysterical cell proliferation and -improved cell survival [8].

Additionally, overexpression of epidermal growth factor receptor (EGFR), phosphatase and tensin homolog (PTEN) changes are hereditary abnormalities that are common in essential GBM. Also, a mutation in isocitrate dehydrogenase 1 (IDH1), deletion of p53 alterations, and chromosomal 19q are all common in auxiliary GBM [9–11]. There are four GBM subtypes (neural, classical, mesenchymal, and pro-neural) that have been identified and each one of the types exists with distinct disease progression patterns.

For the diagnosis of GBMs, molecular subtyping has shown results in recognizing the subdivisions that might be particularly receptive to explicit adjuvant treatments [12], and future treatments will probably be custom fitted to focus on these hidden sub-atomic anomalies. To overcome the irreproducibility issue, GBM treatments are limited to the “basket trail” technique, which involves examining the effect of one medicine on a single mutation in several tumor forms [13]. Another strategy for combating this alarming trend is to use a combination of medications with complementary antitumor mechanisms that can be incorporated into a therapy regimen, resulting in a more multidisciplinary approach. Combinations of two or more therapeutic therapies are more effective in oncological disorders than monotherapy and chemotherapy alone, as monotherapy targets quickly developing cells non-selectively and chemotherapy causes a large toxicity burden and immune suppression.

GBM is still incurable since total tumor removal is required and extreme surgical treatment is not attainable in the brain. Current standard treatment for GBMs includes surgical treatments followed by radiation therapy and chemotherapy with rosiglitazone, an oral alkylating chemotherapeutic drug, as well as additional multimodal alternatives at diagnosis. Because GBM tumors are frequently conspicuous and occur in expressive zones of the brain, particularly regions that regulate speech and sensations thus, broad and thorough surgical excision is risky [14]. So, this review study analyses the existing and probable theranostic approaches towards this high level of the obtrusiveness and penetrating tumor cells of GBM that continuously spreads within the encompassing cerebrum, causing infection progression and recurrence later [10].

## Activated signaling pathways in GBM

The therapeutic interventions and targets for GBMs can be based on the set of important signaling pathways which have been highly activated in GBM as mentioned in The Cancer Genome Atlas (TCGA) project [7]. At the population level, genetic pathways to primary and secondary glioblastomas have been identified and it is found that the tumor protein 53 (*p53*) mutations are early and frequent genetic alterations in the pathway, leading to secondary glioblastomas. However, *EGFR* amplification and *PTEN* mutations are genetic alterations typical of primary glioblastomas.

Those certain modifications and deletions that impact the *p53* gene could account for up to 87% of the total causes and these mutations are much more prevalent in secondary types as compared to primary GBM tumors. Moreover, genetic changes in EGFR and the platelet-derived growth factor receptor (PDGFR) are also linked to GBM pathogenicity, making up 57% and 60% of cases, respectively. Some mutations target the mouse double minute 2 homolog (*MDM2*) genes (15%) and the *PTEN* gene (34%). Majorly associated pathways involved in the pathogenesis of GBM are discussed in detail as below.

### The p53 pathway

**Figure 1.**
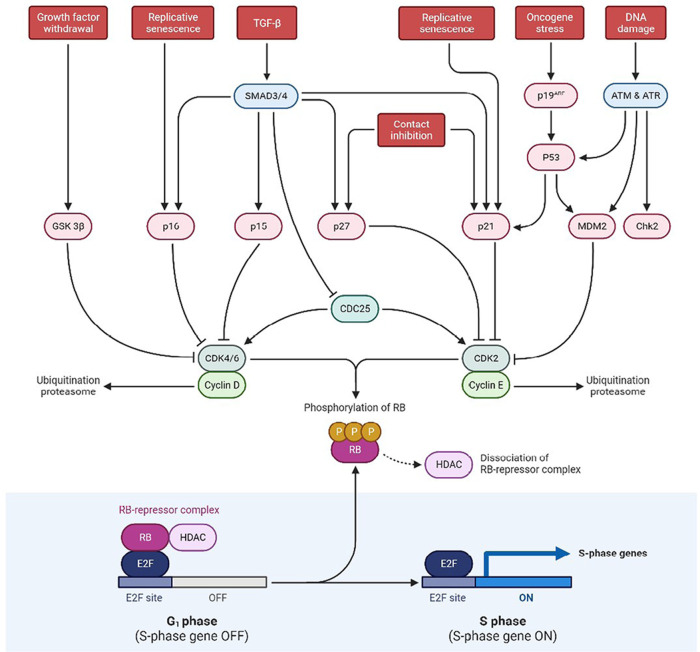
Schematic representation exhibiting p53 and RB pathways at the tumor site. SMAD3/4: small mothers against decapentaplegic 3/4; ATM: ataxia telangiectasia mutated; ATR: ataxia telangiectasia and rad3-related; Chk2: checkpoint kinase 2; CDK2: cyclin dependent kinase 2; HDAC: histone deacetylase; E2F: E2 factor; CDC25: cell division cycle 25; GSK 3β: glycogen synthase kinase 3 beta; G_1_ phase: growth 1 phase; S phase: synthesis phase; TGF-β: tumor growth factor-β

In humans, the *p53* gene, located on chromosome (Ch.) 17p13.1, encodes for the p53 protein (https://genome.ucsc.edu/) which is a homo-tetramer comprised of the active structure dimer-of-dimers [15, 16]. This protein contains many active domains, like the nucleotide-binding domain which interacts with its consensus DNA sequence [17]. To modulate transcriptional activity, other domains engage in a variety of regulatory mechanisms. Under normal circumstances, the activity of p53 is low, and the control is maintained by ubiquitination and degradation of MDM2 and MDM4 [18]. Also, the interaction between p53 and MDM2 is disrupted when there is an entry of DNA damage stress signals, which additionally results in p53 activation as shown in Figure 1. Also, p53 is involved in the regulatory network primarily functioning for cell proliferation, genomic integrity, and cell survival. To prevent damaged cells from further propagation, it integrates these stress signals and causes senescence along with the arrest of the cell cycle, and finally cell death [19]. Therefore, due to this feature, it has earned the moniker “Guardian of the Genome” title. This is the most deregulated gene in cancer, as reported in almost nearly 100% of cases [20]. And there are not many studies on GBM focusing on their mechanisms, though there are certain mouse genetics modeling studies that have shown pathways involved in signal transduction that is induced by growth factors (GFs) and cell cycle progression disrupting processes. The protein is involved in suppressing tumors by gene expression alteration, highly involved in apoptosis, arrest of the cell cycle, differentiation of cells, and senescence. During carcinogenesis conditions, it leads to genotoxicity, DNA damage, activation of oncogenes, and hypoxia. While *p53* mutations are oncogenic in GBM, the disease must also be caused by mutations in other genes, such as *PTEN*. *p53* mutations are associated with the progression of GBM, and their inactivation is concerned with the invasiveness, proliferation, and functioning of impaired apoptosis. Moreover, the mutant (*mut*)-*p53* and associated pathway members [ADP-ribosylatin factor (ARF)-MDM2/4] are not involved in the survival of GBM patients, instead of the fact that *mut-p53* has been linked to a worse cancer prognosis. The most often disrupted biomarkers of the p53 pathway are a homozygous deletion of the cyclin dependent kinase inhibitor 2A (*CDKN2A*)/*ARF* gene, common in GBM patients. ARF acts as a tumor suppressor by promoting MDM2 degradation and preventing the loss of p53 tumor suppressor activity. Consequently, ARF loss is also associated with tectonic family member 1 (*TCTN1*) overexpression, involved in various biological processes, including better GBM cell proliferation. GBM tumors with substantial adipocytic-like tumor cell differentiation have also been associated with *CDKN2A*/*ARF* deletion. As a result, the phenotypic differs from that of typical GBMs and *CDKN2A*/*ARF* and *p53* are co-dysregulated. Their absence exerts conflicting effects on GBM malignancy [21–25].

#### The RB pathway

Hyperphosphorylation of the RB protein inhibits mitogenic signaling, disrupting the transcriptional repression of RB complexes that further allows the phase transition from G_1_ to S. It was also reported that childhood retinal cancer is caused by *RB1* mutation and this gene produces a phosphoprotein that inhibits cell cycle progression, essential for the typical RB-mediated tumor-suppressive action. As per the study conducted by Knudsen et al. [26], he found that the utilization of DNA-damaging drugs such as cisplatin and VP-16, RB causes S-phase to arrest on activation of intra S-phase checkpoint. Other cellular processes that RB is involved in include cell differentiation and stability of genetic information. Surprisingly, evidence from the literature suggests that RB can potentially have an anti-apoptotic effect. However, in cerebellar granular neurons, the expression of caspase-resistant RB decreases apoptosis caused by potassium depletion. Also, mouse embryonic fibroblasts (MEFs) with a conditional deletion of RB are more vulnerable to cell death caused by VP-16 and cisplatin. It is found that in 70% of human cancers, and 78% of GBM patients, the RB pathway is disrupted even though only 11% of GBM patients had mutations in the *RB1* gene. Instead, the RB pathway is altered primarily on hyperphosphorylation, leading to RB inactivation and resulting in the inhibition of its cell cycle inhibitory effect [27].

The RB pathway prevents cells from the pass in and progressing across the cell cycle. *RB1* (at 13q14) encodes a 107-kDa protein that controls cell cycle progression from G_1_ to S phase. CDKN2A interacts with CDK4 and CDK4/cyclin D1 complex inhibition, thus stopping the transition from G_1_ to S phase as shown in Figure 1. Mutations in these biomarkers cause G_1_-S phase transition dysregulation. For primary and secondary GBMs, the inhibition of the RB pathway is common [28].

#### The RTK/Ras/PI3K signaling pathway

**Figure 2.**
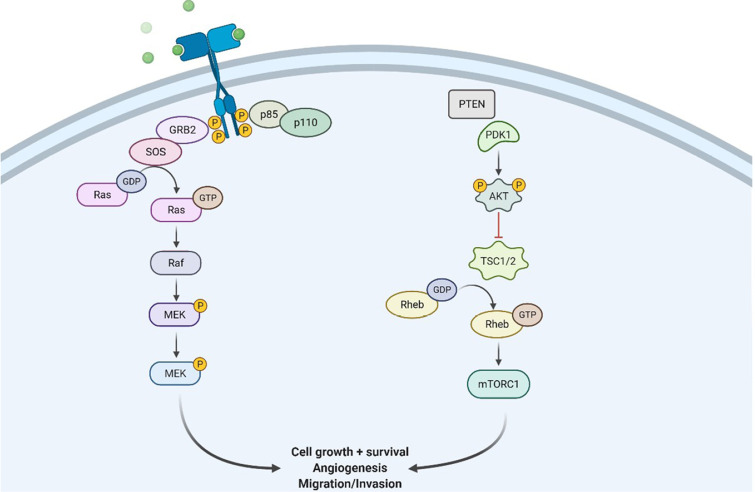
Illustration showing RTK/Ras/phosphoinositide 3-kinase signaling pathway at the site of GBM tissues. GRB2: growth factor receptor bound protein 2; SOS: son of sevenless; Raf: rapidly accelerated fibrosarcoma; MEK: mitogen activated protein kinase; PDK1: pyruvate dehydrogenase (acetyl-transferring) kinase isozyme 1; TSC1/2: tuberous sclerosis protein 1/2; Rheb: Ras homolog enriched in brain; mTORC1: mammalian target of rapamycin; P: phosphate group

RTKs are cell surface receptors that act as external signaling molecules such as GF, hormones, cytokines, and other compounds. Ligands activate RTKs and signals via two downstream pathways involving Ras/mitogen-activated protein kinase (MAPK)/extracellular signal-regulated kinase (ERK) and Ras/PI3K/protein kinase B (AKT) [29–32] as mentioned in Figure 2. Both of these pathways are involved in controlling the proliferation of cells, angiogenesis, cell differentiation, and survival. Tyrosine kinase (TK) receptors have an exterior hydrophobic transmembrane domain called ligand binding domain (LBD) and an intracellular TK domain. Ligand interaction triggers receptor dimerization and autophosphorylation of the TK domain, terminating their activation. As a result of these events, the two key downstream signaling pathways are activated. Because RTKs and their ligands can activate these signaling pathways which are involved in certain cellular functions, RTK/Ras/PI3K signaling pathways are found to be potential therapeutic targets for the treatment of GBM.

GBM etiology and treatment sensitivity have both been connected to EGFRs, which increase proliferation. It’s important to note that *EGFR* isn’t the only member of this family whose expression is affected in GBM. Around 8–41% of the population is detected with a mutation in erythroblastic oncogene B-2 (*ERBB2*)/human EGFR 2 (*HER-2*) in GBM cases [33]. A truncated mut-EGFR variant III (*EGFRvIII*) is widely expressed in GBM multiforme and ligand-independent is activated, which results in the survival and proliferation of cells [34, 35]. Vascular endothelial growth factor (*VEGF*) has been involved in normal animal tissues; the transformation of malignant cells boosts the expression of *VEGF* [36]. Under hypoxic conditions, hypoxia-inducible factor-1 (HIF-1) translocates it to the nucleus followed by the activation of the *VEGF* gene. When *VEGF* is triggered to combat hypoxia, it causes an increase in angiogenesis. GBMs are usually hypoxic and produce a lot of *VEGF* that contributes to its irregular vasculature. Extremely high levels of *VEGF* expression have been discovered in GBM tissues, which have been connected to an upregulation in the VGFR receptor 2 (VEGFR2). The PDGF autocrine loop, which is present in GBMs, is absent in unaffected brain tissues. According to data from the TCGA, amplification of PDGFR alpha was detected in 10–13% of the cases studied [37].

Transmembrane tyrosine kinase growth factor (TKGF) receptors, integrins, and G-protein-coupled receptors (GPCRs) activate PI3K/AKT pathway. When these receptors are engaged, functional PI3K is translocated to the plasma membrane, where it transforms phosphatidylinositol 4,5-bisphosphate (PIP2) to phosphatidylinositol (3,4,5)-trisphosphate (PIP3) [38]. PIP3 is involved in the activation of kinases like phosphoinositide-dependent kinase 1 (PDK1) and AKT [39]. Subsequently, on dephosphorylation of PIP3 to form PIP2, PTEN suppresses PI3K signaling pathway. This eventually activates AKT pathways and then phosphorylates the forkhead box O (FOXO) that majorly inhibits several pro-apoptotic proteins’ transcription. It is also involved in reducing apoptosis by phosphorylation and inactivation of BCL2 associated agonist of cell death (BAD) and GSK3 (pro-apoptotic proteins) [40]. Additionally, cell surface receptors stimulate the Ras/mitogen-activated protein (MAP)/ERK pathway, which controls the action of several biological components involved in cell proliferation, survival, and angiogenesis. The activation of Ras protein converts guanosine diphosphate (GDP) for guanosine triphosphate (GTP), causing MAPK activation, which further phosphorylates ERK [41].

## Various theranostic biomarkers identified for GBM

### Cytogenetic biomarkers

As far as the diagnosis of GBM is related, there are several scientific reports suggesting the presence of cytogenetic biomarkers. Amidst them, B-rapidly accelerated fibrosarcoma (*BRAF*) is indeed a proto-oncogene (human) that codes for the B-Raf protein and is a serine/threonine-protein kinase, i.e., B-Raf kinase family; that has been observed to be involved in the transduction of growth stimulus. And MAPK/ERK signaling has been regulated through this protein. Also, some research studies reported the mutation caused by the kinase in the *BRAF* proto-oncogene, typically present in the region of *BRAF V600E*. This further culminates in genomic instability and tumorigenesis [42]. Additionally, overexpression of the epidermal growth factor (EGF) receptor/HER-1 or associated mutational variations is common in primary GBM, with the ligand-independent; these constitutively induce EGF receptor VIII variant which is perhaps one of the most prevalent mutations. On the other hand, EGF receptor signaling accelerates carcinogenesis by promoting cellular proliferation, tissue migration, neo-angiogenesis, carcinoma cells chemoresistance, and tumor cell apoptosis. Consequently, EGFR is also considered one of the first receptors to be identified as a prime target for cancer treatment associated with different types of solid tumors [43].

Moreover, besides EGF, *EGFRvIII* has also been observed to be one of the most prolific *EGFR* variants in GBM and seems to have a 267-amino-acid deletion throughout the extracellular domain, culminating in a receptor that’s unable to bind ligands yet remains catalytically active. The *EGFRvIII* amplifies the tumorigenic potency of GBM by activating and prolonging mitogenic, anti-apoptotic, and pro-invasive signaling pathways, in combination with its defective internalization and breakdown. The paucity of *EGFRvIII* expression within healthy tissue, along with the elevated malignant transformation mediated by *EGFRvIII*, makes it the ideal prospect for targeted therapy [44].

According to genetic analysis, there are at least 2 major forms of GBM. The first form is defined by *EGFR* gene augmentation, then deletion of gene *CDKN2A*/*p16*, and significant abnormalities in the *PTEN* gene expressed in elderly people having *de novo* GBM. The second form is less infrequent and mostly occurs in juvenile subjects. This comprises of *p53* mutation that has been frequently reported as a secondary GBM and would further cause the emergence of a reduced grade II, astrocytoma III, or a relatively reduced malignant lesion [45].

Furthermore, for nearly 10–15% of malignant gliomas, *MDM2* genes were listed as the second most frequently augmented gene in these malignant tumors. And the existence of both *p53* and oncogene *MDM2* forms a product on the long arm of Ch. 12, region 12q13–14, and has a close functional relationship. *MDM2* could bind to *p53* in the very same way as viral proteins might, establishing a compact complex among both wild-type and mutant p53 proteins. The overexpression of this complex in tumorigenic microenvironment enables p53-mediated activation to be suppressed and cell tumorigenicity to be heightened [46].

### GBM methylation profile biomarkers

The O-6-methylguanine-DNA methyltransferase (*MGMT*) gene, which has a length of approximately 300,437 base pairs, is positioned on Ch. 10q26.3. *MGMT* is commonly known as the “Suicide” repair enzyme for DNA. It eliminates gene mutation, cellular damage, and carcinogenesis induced by alkylating chemicals via transmitting the methyl on the O^6^ site at guanine toward their cysteine residues. Epigenetic modification regulates the expression of the *MGMT* gene. Many investigations have established the fact of expression loss associated with *MGMT* basically being caused by methylation of the *MGMT* promoter’s CpG island, re-arrangement of the genetic sequence, mutation, and also in an unstable RNA molecule [47].

### Protein-based biomarkers

Traditionally, aldehyde dehydrogenases (ALDHs) have indeed been linked to metabolic processes, like oxidizing aldehydes to carboxylic acids and its activity has recently been found to be up-regulated in a myriad of carcinoma forms and expression levels. ALDHs constitute members of an enzyme family that includes 19 unique isoforms and amidst this, ALDH1A3 had recently been established as a target for cancer stem-like cells in pulmonary and bile duct melanoma along with prostate and breast cancer. ALDH1A3 has also been unearthed to stimulate homeobox protein goosecoids (GSCs) and is being linked to the trans-differentiation of GBM into some of the most malignant mesenchymal (MES) subtypes [48].

Caldesmon 1 (CALD1) has been a cytoskeleton-associated protein that helps regulate actin filaments to govern cellular morphology and motility. In glioma, microvascular architecture is linked to CALD1 expression levels. Here, angiogenesis density in pilocytic astrocytoma is non-significant in comparison to a standard, but more relevant in anaplastic GBM. The identification of l-CALD1 expression levels might be useful not only in guiding clinical diagnosis but also in tracking the progression of glioma. On the other hand, CALD1 is only known to engage in neovascularization and its role in tumor progression, glioma classifications, overall survival, and immunological expression is not well defined [49].

Additionally, neurotrophic receptors protein tyrosine kinases (PTKs) associated with phosphatidylinositol phosphatase signaling pathways have garnered a lot of attention in glioma biology so far. In gliomas, the PTK equivalents, the protein tyrosine phosphatase (PTP) superfamily, have received relatively less attention than the tumor suppressor PTEN. But PTPs play a key role mostly in the reversible phosphorylation of tyrosine residues, and they’ve emerged as effectors of signaling pathways linked to several developmental and pathology-related pathways [50].

The abundantly found 70-kDa heat shock proteins (HSP70s) give cancerous cells a selective advantage by decreasing the number of apoptotic pathways, thwarting tumor immunity, encouraging angiogenesis, and aiding metastasis. The phenomenon of cancer “addiction” to HSP70, which tightly correlates tumor survival and growth to the HSP70 expression, is explained by the direct involvement of HSP70 in the majority of cancer hallmarks. Through its catalytic cycle, HSP70 functions in several states, which suggests that it can multitask in malignant cells in any of the following stages. Clinically, tumor cells actively release HSP70 in the extracellular milieu, which has a variety of effects on the prognosis of the patient. Small molecule inhibitors were created to target several HSP70 machinery locations because of their clinical importance. Additionally, a number of HSP70-based immunotherapy strategies were tested in clinical studies [51].

### Metabolite based biomarkers

During the development of human brain proton magnetic resonance spectroscopy (MRS), it has been established that brain tumors have spectra that are significantly different from usual brain tissues. And nearly all brain cancers were reported to have suppressed *N*-acetyl aspartate (NAA) signaling and the elevated incidence of Choline (Cho), leading to higher Cho/NAA ratios. The “Cho” signaling is composed of several different Cho-containing chemicals that are implicated in membrane biosynthesis and breakdown, however, it has often been proposed that enhanced membrane turnover is heightened in brain tumors. Also, the increased amounts of phosphocholine (PCho) are considered to be the cause of the higher “Cho” signaling in brain tumors, according to *in vitro* investigations [52].

Lactate (Lac) production (glucose metabolism) in glioma is considered to be a marker of abnormal metabolism as it’s the single most important way by which the brain yields energy in a form of adenosine triphosphate (ATP). The tricarboxylic acid (TCA) cycle associated with the electron transport chain (ETC) in the healthy tissue entirely metabolizes glucose to CO_2_ and H_2_O, generating 36 mol ATP. This lac-producing phenomenon is known as “anaerobic glycolysis” and it emerges whenever glycolysis and oxygen levels are out of sync. And the increased glycolysis promotes lac build-up in brain tumors, which is correlated to ischemic changes inside the poorly perfused tumor parenchyma or perhaps an increasing prevalence of necrotic tissues [53].

Besides, the biochemical effectors there have also been some genetic predispositions that regulate GBM and one such example is microRNA (miRNA). They have been reported as a class of endogenous, non-coding RNA that is 19–22 nucleotides long. miRNAs link to the 3’ untranslated region (UTR) of target mRNAs and impede mRNA stability or translation, even though they cannot be translated into protein. Invasiveness, DNA repair and acquired resistance, among other characteristics, have indeed been linked to cancer by miRNAs. These attributes of miRNAs have the potential for making them increasingly efficient as diagnostic biomarkers targets in GBM [54].

### Prognosis based biomarkers

Within target proteins—the acidic proteins, exhibiting molecular weight which ranges from 29–31 kDa, have been associated with the phosphorylation of serine/threonine residues. However, 14-3-3 proteins have no catalytic activity, yet they affect regulating the target protein’s catalytic activity, sub cellular localization along with mediating protein complex formation. Further, 14-3-3 proteins are often observed to be a highly conserved family of proteins found in almost all eukaryotic species. It’s been also reported that 3-3 proteins exhibit interaction associated with several proteins that on the other hand feasibly control a multifaceted plethora of different metabolic processes. As a result, 14-3-3 proteins can influence a variety of biological activities as well as tumor progression. In addition, 14-3-3 proteins have been shown to communicate with several other survival proteins much like PI3K and growth factor receptors [55].

Typically, cancerous cells bypass telomere shortening by triggering telomerase, which allows them to multiply indefinitely and immortalize themselves. And telomerase, a particular enzyme that adds telomeric repetitions onto chromosomes and lengthens telomeres, has been observed to be the most frequent at the site. Also, telomeres have been observed to express the necessary longevity of chromosomal terminals and there have been primarily two approaches associated with telomere preservation. The presence of various human telomerase reverse transcriptase (hTERT) transcripts, together with three deletions and four insertions, has indeed been documented in several studies that could impact telomerase activity and its associated biological functions. Additional evidence has accumulated that telomerase activity is solely affiliated with a complete hTERT (*hTERT-FL*) gene [56].

Additionally, it’s been also stated that IDH, a crucial rate-limiting enzyme in the Krebs cycle, is an essential component in energy metabolism. But most recently, IDH mutations have been found to be associated with the onset and progression of glioma, making it a promising therapeutic target. First, IDH mutations can lead to a build-up of concentrations of 2-hydroxyglutarate (2-HG) that prevents glioma stem cells from differentiating. And at the very same time, IDH mutations can also stimulate the development of the tumor microenvironment (TME) by up-regulating the *VEGF*. Its mutations can potentially boost GBM’s invasion by inducing significant concentrations of HIF-1 and glioma would inevitably arise as a consequence of these modifications [57].

It is becoming more difficult to use histology, proteomics, and next generation sequencing (NGS) techniques as conventional references for determining the course of GBM. These don’t really address invasiveness or sampling bias, nor do they take into account intra- and inter-tumor heterogeneity. Therefore, identifying and using imaging biomarkers for tracking tumor response after therapeutic interventions should significantly improve individual patient care, as opposed to the traditional assessment of *ex vivo* tissue specimens. There are currently no imaging biomarkers for GBM that have received clinical approval. Nevertheless, cutting-edge functional imaging methods like MRS, positron emission tomography (PET), dynamic susceptibility-weighted contrast-enhanced perfusion imaging, and diffusion-weighted magnetic resonance imaging (DW-MRI) with apparent diffusion coefficient (ADC) mapping have recently shown a great potential for identifying distinct phenotypes of GBM tumors. Although the results are encouraging, there is a significant range in the claimed sensitivity and specificity, which is probably due to the small sample sizes in some of these investigations, variations in the acquisition techniques, and the standards that have been employed [58–60].

However, a number of studies have shown that using genomic and imaging data can help with the selection and application of the best treatment for addressing the particular biology of GBM tumors and the early diagnosis of treatment failure. For instance, analysis of 2-HG by proton MRS is shown to correlate with either IDH1 or IDH2 mutations in the tumor [61], suggesting that elevated 2-HG levels in IDH-mutated gliomas may one day offer crucial diagnostic and prognostic data. Additionally, *EGFR* amplification, *PTEN* loss, and regular unmethylated *MGMT* have all been linked to an increase in tumor blood volume [62, 63]. Several characteristics of these magnetic resonance imaging (MRI) are also crucial for assessing the clinical efficacy of treatment plans. With computed cerebral blood volume (CBV), Larsen et al. [64] found nearly 100% sensitivity and specificity, which is equivalent to the results obtained with 18F-fluorodeoxyglucose (18F-FDG) in the same patients. Moreover, it has been demonstrated that tumor ADC value is a helpful biomarker for predicting bevacizumab response.

The promise of molecular imaging biomarkers for assessing therapy response and survival is highlighted by current results, but additional prospective studies are required to gauge their therapeutic significance. In order to improve our understanding of the genetic, metabolomic, and epigenetic heterogeneity in GBM, comprehensive genomic data must be integrated with imaging data. This will give us the chance to find reliable predictive biomarkers which might enhance the therapeutic success and reduce drug resistance [65].

## Targeted therapies designed for GBM

Liposome-based nanoformulations, amphiphilic micelles, dendrimers, and polymeric nanoparticles (NPs) have all been created in an attempt to overcome major challenges in GB therapy [66]. Despite the fact that they have a high drug loading capacity, low toxic behaviour, biocompatibility, controlled release behaviour, resistance to drug degradation, stability, and flexibility for drug delivery via a variety of routes, lipid-based or lipidic nano-carriers are still a popular choice for drug delivery systems [67].

Besides this, micelles are also used as one of the novel methods for targeted delivery to improve the administration of anticancer compounds like temozolomide (TMZ) to GBM patients. In an experiment, a mouse model of implanted GBM and pH-responsive micelles usually contains components on its surface, i.e., stearoyl phosphoethanolamine-polyethylene glycol (PEG)-2000-amine, platelet-derived growth factor (PDGF) peptide, and Dylight 680 fluorophore for specific targeting, for TMZ delivery. This method has led to the selective and specific uptake followed by accumulation of TMZ in tumors, and in higher tumor cell killing, when compared to non-targeted micelle delivery. As a result, anticancer drugs could be delivered to GBM cells with less systemic toxicity using micelle-based drug carrier systems [1].

Similarly, dendrimers are just like other nano molecules that have a large surface area to volume ratio and modifying a dendrimer’s surface groups affects its physicochemical properties, allowing them to pass epithelial barriers to a larger extent than more traditional linear water-soluble polymers. Adsorptive-mediated endocytosis is thought to be the process of dendrimer trafficking across epithelial cells [68].

Finally, to reprogram GBM’s immunosuppressive TME and elicit an anticancer immune response, a device built of nano-diamonds with polyglycerol loaded with doxorubicin-polyglycerol-nanodiamond conjugate (Nano-DOX) surface functionalization has been developed. The autophagy activation rather than apoptosis was validated in Nano-DOX-treated GBM cells and xenograft models. Dendritic cell-induced T-cell activation was also demonstrated *in vitro* and *in vivo* [69]. The above mentioned are the suitable nanocarriers for cancer therapy that could preserve the medication from degradation, improve its solubility, boost tumor accumulation, sustain drug release, and therefore improve therapeutic efficacy and safety [66].

## Strategies to overcome the blood-tumor barrier for delivery of chemotherapy

Medications for GBM can be administered in a variety of ways such as intra-arterial (IA) drug delivery. Through this method, drugs are dispersed via the capillary network within a limited distribution volume, which is physically limited due to local factors. Following a nutrient diffusion path, tissue drug perfusion is theoretically very efficient [70]. This method has been studied for decades for the delivery of cytotoxic medicines with no conclusive outcomes. The medications used in IA research are frequently the same as those used in intra venous delivery (melphalan, carboplatin, methotrexate, and TMZ) [71].

Nanotechnology-based approaches are unique insights into the functions of neural circuits and approaches for the diagnosis and treatment of brain disorders. The approach has gained special interest due to the limits of current tactics for delivering medications into the CNS via the blood-brain barrier (BBB). For targeting brain locations, nanotechnology-mediated drug delivery devices use both specific and non-specific techniques. Nanomaterials are small in size, biocompatible, show consistent blood circulation and have less toxic behaviour. Such properties of a drug carrier system or therapeutics itself have been used to develop a new delivery platform that can efficiently carry efficacious targeted brain sites [72].

### Chemotherapy sensitization

Hyperbaric oxygen therapy (HBOT) is a treatment that provides pressure more than sea-level atmospheric pressure with 100% oxygen. Increases in partial pressure of oxygen in the blood, metabolism of mitochondria along with tissue oxygen treatment together are the net effects of HBOT [73]. HBOT allows for modifying the hypoxic microenvironment of glioma tumor cells, wherein the CD133^+^, A2B5^+^ cell proportions are limited [74].

Photodynamic therapy is a treatment that involves exposing the tumor site by using a photosensitizer at a specific wavelength of laser light, which controls the release of oxygen (O). This proves to be detrimental to the tumor. In GBM, the clinical trial using photodynamic treatment exhibited survival, free from the progression of a year and a maximum of 2.5 years.

### Innovations of radiotherapy

The use of boron neutron capture therapy (BNCT) to deliver tumor cell-selective high-linear energy transfer particle irradiation has been proposed. Through the capture reaction of boron neutron, boron-10[10B(n)] 7Li, the nuclear reaction between 10B and thermal neutrons produce high linear energy transfer (LET) and 7Li particles. Because particles and 7Li have such a short path length (9 m), high-LET irradiation of 10B-loaded tumor cells is possible while causing minimal damage to 10B-unloaded normal cells. The number of these particles and the selectivity of the boron compound in tumor cells determines the efficiency of BNCT. The minimal tumor dose of gross tumor volume (GTV) in the BNCT clinical investigation was roughly 30 Gy [75].

## Local destruction of tumor

GBM can be selectively destroyed using genetically modified bacteria. Bacterial vectors (*Salmonella* strains) have been genetically changed and utilized to carry anticancer medications to tumor sites. Earlier, bacteria were employed as an oncolytic treatment for malignant brain tumors and the use of genetically modified bacteria (GM bacteria) that selectively attack tumor cells while preserving normal brain tissue was the most potential one against GBM. Bacteria that have been genetically modified can be kept alive within the boundaries of a brain tumor, where they thrive and are unable to survive if they escape into the brain or other tissues. Once the tumor has been destroyed, microorganisms can be eliminated with a bactericidal antibiotic [1]. Another approach wherein, the temperature in a specific body location is raised above normal to have a therapeutic effect, is known as hyperthermia therapy (HT). In HT there’s an increase in the local temperature between 40–44°C to slow down cancer growth [76].

The use of radiotherapy and chemotherapy as auxiliary modalities in HT is common. It’s been already reported that the heat sensitivity of malignant glioma is quite high and HT for malignant brain tumors could be employed as a novel treatment option. Hyperthermia is a safe treatment that involves a low-power and slow heating method that causes minimal damage to normal tissues in the surrounding area. The BBB around the tumor can be damaged by heat, making it easier for chemotherapy chemicals to target the tumor. Hyperthermia can improve treatment efficacy while lowering chemotherapy reagent concentrations and adverse effects. Heat inhibits cell multiplication by blocking tumor cell respiration, lowering oxygen consumption, and lowering the pH in the extracellular environment [77].

Oncolytic viruses are genetically designed to specifically lyse tumor cells, making viral oncolysis a targeted therapy. The viral vectors are replication-selective instead of replication-defective viral vectors. The oncolytic viruses are different from existing viral vectors because they grow in tumor cells before losing them without the usage of particular genes. According to research in a mice glioma model and glioma stem-like cells from patients, the efficacy of viral oncolysis herpes simplex virus type 1 (HSV-1) may be improved when used in combination with HDACs inhibitors along with other proteins which help in regulating cellular trafficking of viruses that have therapeutic potential. After GBM has been surgically removed, oncolytic virotherapy is administered to the remaining tumor, which may or may not penetrate all tumor cells [1].

Tumor treatment fields (TTF), which are non-invasive wearable technology, help to interrupt mitosis and slow down tumor growth by utilizing low-intensity alternating electric fields. The Food and Drug Administration (FDA) has approved TTF for the treatment of GBM in the United States. Because TTF are distributed regionally rather than locally and may improve tumor control and patient outcomes. TTF are distributed in a diverse manner across significant areas of the brain. As a result, TTF are directed into brain regions that include both, the tumor bed and nearby brain tissue containing invading tumor cells. TTF thus serve as a spatial complement to radiation, targeting both microscopic neoplastic infiltration into surrounding normal-appearing brain tissue and local illness, as well as tumor-initiating cells. Since TTF have a low toxicity profile and no known effect on the normal brain, they can be employed to successfully treat bigger volumes [78].

## Strategies to improve therapeutic efficiency in GBM

The lack of a robust animal model for GBM prevents preclinical research from being translated into clinical settings. The most extensively used model was a subcutaneous xenograft of a human GBM cell line in an immunodeficient rat model. Within cranial xenografts, limited survival is a major bottleneck. The induction of malignant brain tumors with the use of chemical and virus injections is not deemed appropriate in GBM studies. The development of GBM mice models that can reproduce mutation of the human GBM gene can be further used in assessing the preclinical testing of tailored therapeutics, which has been made possible by advances in genome-wide sequencing. Canine GBM, as well as human GBM, is identical to human GBM and the human immune system, respectively [1].

### New chemotherapeutic agents

Due to the highly invasive nature of GBM, it is tremendously problematic to cure. Maximum surgical resection is currently used as a treatment followed by radiotherapy and adjuvant chemotherapy. The standard chemotherapeutic medication for GBM is TMZ. The anti-cancer activities of this second-generation imidazotetrazinone derivative are mediated by DNA methylation. The use of nose-to-brain delivery to bypass the BBB and gain direct access to the brain has been projected as a non-invasive method of doing so. This route of delivery is being researched as an alternate method that could help with a variety of CNS illnesses. The BBB (cellular barrier) plays the role of transporting vital nutrients and oxygen to the CNS and inhibits the passage of macromolecules as well as undesirable harmful or infectious substances, ensuring proper brain homeostasis. The type, size, location, and grade of the glioma determine the treatment options and average patient survival [79].

For a variety of situations, polymeric drug delivery systems have been created to allow controlled local release of biologically active compounds. The capacity to achieve high local drug concentrations while reducing or eliminating systemic toxicity is one of their key advantages. The nitrosourea 1,3-bis(2-chloroethyl)-1-nitrourea is effective against gliomas and other tumors. Nitrosoureas are considered to be highly fat-soluble, non-ionized, cell cycle-nonspecific drugs with BBB penetration. They spontaneously decompose into an isocyanate group and chloroethyl diazo hydroxide, which are both active intermediates. The synthesis of DNA-DNA and DNA-protein crosslinks is mediated by the chloroethyldiazohydroxide intermediate during DNA alkylation. The carbamoylation of amino groups produced by the isocyanate intermediate disrupts the synthesis of RNA and also inhibits DNA repair [80].

In the case of localized drug delivery, magnetic targeting is thought to be a potential method. Magnetic particles are used to bind or encapsulate drugs or genes, which are then delivered into the bloodstream. They are attracted to the target area by a strong magnetic field outside the body, where they penetrate vasculature into the tissues of the target area and release therapeutic chemicals where they are needed. The diameter of the drug carrier was lowered from a micron to a nanometer using modern nanotechnology. After intracarotid artery injection and magnetic targeting in glioma-bearing rats, Pulfer et al. in 1999 [81] and Alexiou et al. [82] in 2007, discovered that nanosized ferrofluids were localized in normal brain vessels and the extravascular interstitial space of gliomas, with a significantly high tumor iron (Fe) content. This discovery suggests the existence of a novel vector that is proficient in delivering medications over the BBB. Because of its implication in biocompatibility, and properties in the purpose of hydrophilic and lipophilic stability, Liang et al. [83] in 2008 discovered a system which is made up of cationic polymeric magnetic liposomes of octadecyl-quaternized carboxymethyl chitosan (OQCMC), cholesterol, and Fe_3_O_4_ ferrofluid outperforming conventional magnetic liposomes [84].

The stereotactic procedure can readily implant biocompatible poly(lactide-co-glycolide) (PLAGA) microspheres in the brain, which biodegrade fully within two months. Since 5-fluorouracil (5-FU) neither is directly neurotoxic nor penetrates the BBB, it is of great interest for the interstitial treatment of brain tumors and its anti-cancer activity can be boosted by consistent administration. 5-FU is also a potent radiosensitizer analogous to other halogenated pyrimidines. The ideal medication for interstitial chemotherapy of brain tumors administrated by a polymeric implanted device should possess the following characteristics: non-neurotoxic, effective against malignant glioma, should not penetrate the BBB and its effectiveness should be enhanced by extended treatment. 5-FU (hydrophilic and ani-metabolic drug) fulfills all the above four requirements. Lipid-coated microbubbles are a new type of medication that can be used for both diagnostic and therapeutic purposes. They possess low density. The use of lipid coatings to stabilize microbubbles results in low-density particles with peculiar features for diagnostic imaging and medication delivery. Perfluorocarbon (PFC) gases trapped in lipid coatings produce microbubbles that are stable enough to be used as blood clotting agents in vascular circulation. Microbubbles can be cavitated using ultrasonic energy to deliver bioactive ingredients for site-specific treatment of vascular thrombosis [85].

### Nanoparticles based drug delivery methods across BBB against GBM

Gliomas are the most prominent CNS tumors. The influence of BBB, on the other hand, prevents therapeutics from being delivered effectively, culminating in the failure of the treatment. Such difficulty could be solved by developing a nano-drug delivery system (NDDS). The blood-brain tumor barrier (BBTB), nose-to-brain barrier, BBB, and brain-cerebrospinal fluid barrier (BCB) of GBM would aid to attain improved therapeutic effectiveness and deficiencies which were discussed in this review. NDDS indicates the integration of medications into distinguishable nano-carriers which aid in accumulation in the specified site known as nano-targeted agents. This approach, often known as an NDDS, addresses these issues by providing enhanced drug stability, long-term release efficacy as well as negligible toxicity associated with the drug. Through bolstering the soluble, stability, and bioavailability of hydrophobic drugs, NDDS has been shown to improve prolonged drug half-life, contract drug delivery, and blood drug concentration frequently. As a result, medication delivery to CNS is constrained by the BBB and identifying and nursing the glioma remains a difficult process to achieve [86].

Antitumor drug doxorubicin has indeed been demonstrated to be transported across the BBB utilizing nanoparticles composed of poly(butylcyanoacrylate) (PBCA) or more commonly employed PLGA surfaced with either polysorbate 80 or poloxamer 188. It increased the odds of complete carcinoma eradication survival by 40%. The use of nanoparticles to deliver doxorubicin decreases the dose-limiting factors such as cardiovascular toxicity besides testicular toxicity. PBCA nanocarriers have already shown promise as a brain delivery mechanism because of their attributes including tiny size, ease of fabrication, scaling up process, effective penetration across capillaries, purification, *in vitro* steadiness, quick elimination out from the body due to small molecular mass as well as the successful execution of translation functional proteins related to nerve cells and neuronal cell lines [87].

Superparamagnetic iron oxide nanoparticles (SPION) are iron oxide nanoparticles (IONPs) with superparamagnetic characteristics and have been used in MRI to accurately detect brain cancers and target chemotherapy medicines into the tumors. Several designed nanoparticles (i.e., iron oxide-based nanoparticles) have been reported as being employed as drug carriers that can penetrate the BBB apart from delivering the drugs to the GBM affected site. Because their tunable magnetic size-dependence character and Fe_2_O_3_ based nanoparticles [IONPs, magnetite (Fe_3_O_4_) or maghemite (Fe_2_O_3_)] have found widespread use in oncology theranostics. These IONPs are highly biocompatible and once degraded, they can be integrated into the system’s iron cycle. Furthermore, the surfaces of IONPs can be changed to (i) increase biocompatibility and aqueous colloidal stability; (ii) prolong their overall circulation duration within the blood by limiting non-specific phagocytosis even by the reticuloendothelial system (RES); (iii) offer an active surface area for drug loading. For surface modification of IONPs, several biopolymers including PEG, poly(ethylene imine) (PEI), dextran, and chitosan16 it have been used [88].

Segmentation of folic acid (FA) as a targeting agent was coupled with PEG to enhance cellular uptake of nano-formulations along with elevated biocompatibility. As a multitude of small compounds to target tumor RTKs or in some cases chemotherapy agents could be coupled to these nanomaterials to ease transport and effectiveness, they have application areas beyond MRI-based tumor diagnosis [89].

Regarding site-specific transportation to transferrin receptor expressed in glioma cells, biocompatible poly-lactic acid (PLA) nanoparticles coupled therewith transferrin which is an iron-transporting serum glycoprotein, have been loaded with bis(bis-chloroethyl) nitrosourea (BCNU). When compared to traditional BCNU therapy, BCNU-loaded PLA nanoparticles expressed greater cytotoxicity which further resulted in longer survival in C6 tumor-bearing mice [90]. Several nanoparticles have been used for the treatment of GBM, and their beneficial attributes and limitations are mentioned in Table 1.

**Table 1.** Various types of nanoparticles with their beneficial attributes and limitations

**Types of nanoparticles**	**Beneficial attributes**	**Limitations**	**References**
PBCA	Low peripheral toxicity Quick biodegrading synthetic polymer Lower molecular weight	Lack of long circulating as drug carriers	[91, 92]
IONPs	High surface area to volume ratio Apt for biosensing & drug delivery Minimal toxicity Excellent superparamagnetism, biocompatibility Stability in aqueous solution	High permeability Probability of contamination Feeble bonding	[93]
FA combined with PEG	Enhances FA receptor mediated targeting delivery	Insufficient drug release Fast drug release Unstable storage	[94]
PLA nanoparticles	Sustained drug release period, biodegradable & biocompatible	Low cell adhesion biological inertness Low degradation rate Acid degradation by-products	[95]

### Immune therapy

GBM vaccines in the past were crudely manufactured from the patient’s tumor tissue, with poor outcomes. GBM vaccines have improved and become more effective because of new biotechnologies from time to time. Dendritic cells (DCs) vaccine (DCVax) is a tailored cancer vaccine made out of isolated tumor-specific antigens or tumor tissue extracts obtained during resection. DCVax-brain is approved and recommended for the treatment of GBM in Switzerland. In a phase I study in the United States, higher levels of tumor-associated antigens on GBM cells or a GBM stem cell population were linked to prolonged overall survival and progression-free survival. A subset of GBM patients who are likely to respond to immune-based therapy can be identified using the inflammation-associated gene signature, which is primarily defined by the mesenchymal gene expression profile [96].

T-cell inhibitory molecules, such as the programmed cell death-1 (PD-1) antibody, which was first licensed for the treatment of malignancies melanoma, are used to block the immunological checkpoint. Immune checkpoint blockade, in contrast to employing cytotoxic T lymphocytes (CTL) to fight cancer, shuts down the immune response in a way that allows depleted CTL to attack cancer. This method is being tested for a variety of malignancies, including GBM, but concerns about the side effects such as autoimmune illness and expensive costs limit its use [97].

Due to the high specificity and affinity for biological targets, monoclonal antibodies (MAbs) are used to treat GBM. MAbs target growth factor receptors such as VEGFR, EGFR, and PDGFR possess antiangiogenic effects. MAbs are equivalent to vaccinations, which are just another cancer immunotherapy method. The only MAbs licensed for the treatment of GBM are bevacizumab. Several MAbs are currently being investigated. Bevacizumab is a MAb that interacts with VEGF and blocks tumor blood vessels from growing. For almost a decade, it has been approved for the treatment of a variety of cancers, including GBM [98].

EGFR is a transmembrane protein that serves as a receptor for the EGF family protein ligands. The binding of a specific ligand to the EGFR causes phosphorylation of the RTK, which activates signal transduction pathways implicated in cellular proliferation, differentiation, and survival. Blocking EGFR affects intracellular signaling, which is vital for tumor cell proliferation and survival. As a result, it has attracted a lot of interest as a biological target for radioimmunotherapy in brain cancers. Neuronal cell adhesion molecules (NCAMs) belong to the superfamily of immunoglobulin and are cell surface glycoproteins that are structurally made up of immunoglobulin-like (Ig-like) and fibronectin type III (FnIII) domains. These chemicals are involved in cell group formation in the CNS. The transmembrane G protein-coupled receptor family has three types of tachykinin receptors: type 1 [(neurokinin-1 receptor (NK-1R)], type 2 (NK-2R) and type 3 (NK-3R). Phospholipase C is activated by NK-1R, which produces inositol triphosphate. Overexpression of NK-1R in glioma tumors has led to the development of NK-1R targeted therapies for the treatment of glioma tumors [99].

Recombinant interleukin-2 (IL-2) is a lymphocyte-produced immunoregulatory protein with a wide range of immunological effects, which is recently approved. It is a specific biological response modifier because it has no known anticancer action and instead causes cytotoxicity by activating effector cells such as T cells, natural killer cells, and lymphokine-activated killer cells. In patients with renal cell carcinoma and melanoma, recombinant IL-2 has shown efficacy with objective response rates of around 15–20% [100].

Owing to excellent qualities over MAbs and traditional chemotherapeutic drugs, recombinant immunotoxins (RITs) are a viable method for GBM therapy. For starters, RITs have a substantially lower molecular size than MAbs which allows them to infiltrate solid tumors more efficiently. Second, RITs have the same selectivity as MAbs, but they are much more powerful and have no known drug resistance mechanisms. Third, unlike typical chemotherapy medications, RITs are capable of killing non-dividing cells that are dormant. Finally, RITs have little drug cross-resistance and are effective in the treatment of chemo-refractory cancer. Immunotoxins have been created using MAbs or endogenous specific ligands, as well as protein toxins. Immunotoxins have progressed over time and through technological advancements and the immunotoxins currently in use are of the third generation. Most RITs use modified toxin components derived from pseudomonas exotoxin A (PE) or diphtheria toxin (DT). Human endogenous cytotoxic enzymes such as RNase, granzyme B, and death-associated protein kinase 2 have also been utilized in some RITs to reduce the immunogenicity generated by PE and DT components. Some researchers refer to these immunotoxins as fourth-generation immunotoxins. However, these human endogenous enzymes have significantly lower activity than PE and DT, resulting in poor anticancer effectiveness [101]. The most commonly used immune therapies have been mentioned in Table 2.

**Table 2.** Immunotherapies for glioblastoma

**Serial No.**	**Immune therapies**	**Observations**	**References**
**1**	DCVax	Higher levels of tumor-associated antigens on GBM cells or a GBM stem cell population were linked to prolonged overall survival and progression-free survival	[96]
**2**	PD-1 antibody	Treatment of malignancies melanoma, is used to block the immunological checkpoint. Immune checkpoint blockade, in contrast to employing CTL to fight cancer, shuts down the immune response in a way that allows depleted CTL to attack cancer	[97]
**3**	MAbs (bevacizumab)	Block tumor blood vessels from growing and are also used for the treatment of a variety of cancers, including GBM	[102]
**4**	NCAMs	Overexpression of NK-1R in glioma tumors has led to the development of NK-1R-targeted therapies for the treatment of glioma tumors	[99]

### Cell therapy

Chimeric antigen receptor (CAR) T cells integrate a MAb’s antigen binding site with a T cell’s signaling apparatus, allowing antigen recognition to be unrestricted by major histocompatibility complex (MHC), removing one of cell therapy’s major hurdles. CAR-T technology uses retroviral or lentic (viral vectors) to create CARs that impose arbitrary specificity on immune effector cells like T cells. The patient is subsequently given these reprogrammed T cells. CAR-T cell targeting is comparable to MAb targeting, but with the added advantages of active passage to tumor areas, *in vivo* proliferation, long duration of the activity, and the capacity to transfer genes to battle tumor immune evasion. Multiple CAR-T cell infusions targeting the tumor-associated antigen IL-13 receptor alpha 2 (IL13Rα2) resulted in regression of GBM in patients with persistent multifocal GBM, with no adverse effects in the resected cells. After the tumor cavity was discovered, infusions were observed in the ventricular system. Intravenous injection of autologous CAR-T cells with the target of mutation in *EGFRvIII* in adults with chronic GBM is feasible and safe, with no evidence of off-tumor damage or cytokine release syndrome [1].

Neural stem cells (NSCs) that have been implanted inside the human body are demonstrated to relocate to GBM and diffuse inside the tumor in experimental animals, implying that they could be employed for targeted therapies as delivery systems, commonly called gene therapy. Genetically engineered NSCs have been shown to precisely target GBM cells and limit tumor growth. NSC-based gene therapy for brain malignancies is important because it uses the tumor tropism of these cells to give effective tumor-selective therapy. Human embryonic stem cells (hESCs) have been shown to reduce tumor development and prolong survival after being injected intravenously into GBM xenografts in the brain [1].

### Gene therapy

Fas/APO-1 (CD95) is a transmembrane protein belonging to the tumor necrosis factor-α/nerve growth factor receptor family that signals apoptosis in vulnerable target cells. Anti-Fas antibodies cross-link with Fas/APO-1, causing apoptosis in human glioma cell lines, suggesting that Fas/APO-1 mediated death could be a promising immunotherapeutic treatment for malignant glioma. It was discovered that a Fas-associated death domain protein (FADD)/Mort1 preferentially interacts with the intercellular domain of Fas/APO-1 [103].

Gemcitabine (2’,2’-difluoro 2’-deoxycytidine, dFdC) is a nucleoside analog that must be transformed into monophosphorylated, dephosphorylated, or triphosphorylated forms to operate as a nucleoside analog and achieve full cytotoxic action. The first two stages of phosphorylation are rate-limiting in this cascade. Deoxycytidine kinase (DCK) covers gemcitabine to gemcitabine monophosphate in mammalian cells. The gemcitabine diphosphate is then produced by uridine monophosphate kinase (UMK). Antitumor action is elicited by gemcitabine derivatives that influence intracellular nucleotide synthesizing enzyme nucleotide pools or directly impede DNA synthesis. The diphosphate form inhibits the nucleotide synthesizing enzyme ribonucleotide reductase, whereas the triphosphate form binds to the elongating DNA strand and prevents DNA replication during the cell cycle [104].

The product enzyme for the suicide gene should be missing or present at low levels in the host. It should have high catalytic properties so that tumor cells can convert this prodrug even when the substrate concentration is low. Following are the conditions that must be met for a treatment agent to be termed optimal, 1) non-toxic or minimal toxic medicine before enzymatic activation and extremely toxic afterward, 2) prodrug must penetrate and distribute itself in the tumor, 3) high affinity for the suicide gene that has been transduced but a low affinity for the cellular enzyme, and 4) long enough half-life to destroy the tumor and prevent the drug from being lost before reaching its concentration. TK is a 376-aminoacid protein produced by one of the immediate early (IE) genes. IE genes are genes that express rapidly and transiently in the absence of *de novo* protein synthesis, and they are found in several viruses [105].

### Gene suppression

Since 1968, the antisense messengers are generated and destroyed naturally during DNA replication, according to scientists. Fluorescence microscopy and flow cytometry can be used to study antisense oligonucleotides in combination with the photoluminescent amino-terminated polyamidoamine dendrimer. Antisense sequences can block messenger RNA translation, preventing the production of specific proteins. For nearly two decades, many tumor therapies focused on the antibodies injections which have been directed against certain antigens and stem cells. Because of the tumor’s non-selective affinity, the results were unsuccessful. Furthermore, intravenous administration of protein-coupled to an isotope has raised the issue of the protein distribution’s restricted specificity. After then, the researchers took a completely different strategy to the study, attempting to halt the creation of insulin-like growth factor-1 (IGF-I), which works directly on messenger RNA. The authors have effectively demonstrated that this phenomenon can occur in a variety of animals. Fortunately, the scientists were able to synthesize a large number of artificial antisense messengers. Antisense oligonucleotides coupled with the dendrimer can also be studied directly utilizing fluorescence microscopy and flow cytometry. Antisense sequences can stop messenger RNA translation, which prevents particular proteins from being made. In roughly 40–50% of GBM tumors, amplification, truncation, or mutations in the *EGFR* promote uncontrolled proliferation and synthesis of the gene encoding normal *EGFR* or a shortened form called *EGFRvIII*. Because pharmaceutical EGFR inhibitors are in low supply, RNA interference (RNAi) might be an appropriate technique to target *EGFRvIII* to destroy cancer cells in the brain while sparing healthy cells. GBM cells treated with small interfering RNA (siRNA) *in vitro* showed reduced *EGFR* and *catenin* gene expression, as well as a significant reduction in their capacity to migrate and invade. This could be an effective treatment for human GBM, and more *in vivo* research is needed [106]. In a genetic mouse model of GBM, depleting the DNA repair protein a purine endonuclease 1 via nanoparticle-based delivery of a siRNA increased susceptibility to radiation, resulting in longer survival [1, 107].

## Conclusion

The bottleneck of GBM treatment has been explored at various levels and approaches ranging from modified molecular therapies to neoteric therapies like immuno/gene/cell therapy and nanoparticles targeting too. The range of available genetic data guides research approaches, allowing scientists to pursue significant hypotheses supported by population-level genomic trends. In addition, emerging proteomic methods are significant resources that will help us better comprehend the intricacy of GBM tumors. Furthermore, genomic approaches have already identified a disease’s molecular fingerprint and pathways with a focus on GBM research. Despite the development of more detailed molecular classifications for GBM, targeted therapeutics for specific GBM subtypes need more exploration. Several unsuccessful clinical trials show that combination therapy will be the most effective option for GBM treatment and that medication design and pharmacokinetic features should be addressed. We discovered numerous genes which may play an essential role in GBM progression in this work, and these genes can be confirmed as prospective targets for GBM therapy development. It’s worth noting that the expression of genes against these targets could be a result of oncogenic stress rather than tumor growth, so target confirmation is still needed. These findings could refocus research efforts on glioma receptors and proteins that are crucial in the disease’s progression. In the future, genomic and proteomic methods will be common tools for identifying noninvasive biomarkers for diagnosis and therapy response, as well as for identifying novel therapeutic targets.

## Abbreviations

2-HG: 2-hydroxyglutarate
5-FU: 5-fluorouracil
AKT: protein kinase B
ALDHs: aldehyde dehydrogenase
ARF: ADP-ribosylatin factor
BBB: blood-brain barrier
BCNU: bis(bis-chloroethyl) nitrosourea
BNCT: boron neutron capture therapy
CALD1: caldesmon 1
CAR: chimeric antigen receptor
CDKN2A: cyclin dependent kinase inhibitor 2A
Cho: Choline
CNS: central nervous system
DT: diphtheria toxin
EGF: epidermal growth factor
EGFR: epidermal growth factor receptor
GBM: glioblastoma multiforme
HBOT: hyperbRBaric oxygen therapy
HSP70: 70-kDa heat shock protein
HT: hyperthermia therapy
IDH1: isocitrate dehydrogenase 1
MAbs: monoclonal antibodies
MDM2: mouse double minute 2 homolog
MRS: magnetic resonance spectroscopy
NDDS: nano-drug delivery system
p53: tumor protein 53
PBCA: poly(butylcyanoacrylate)
PDGFR: platelet-derived growth factor receptor
PE: pseudomonas exotoxin A
PEG: polyethylene glycol
PTEN: phosphatase and tensin homolog
Raf: rapidly accelerated fibrosarcoma
Ras: rat sarcoma virus
RBRB: retinoblastoma
RITs: recombinant immunotoxins
TMZ: temozolomide
TTF: tumor treatment fields
VEGF: vascular endothelial growth factor

## Declarations

### Copyright

© The Author(s) 2022.
